# Non-neuronal Role of Acetylcholinesterase in Bone Development and Degeneration

**DOI:** 10.3389/fcell.2020.620543

**Published:** 2021-01-28

**Authors:** Xiaohe Luo, Marianne Lauwers, Paul G. Layer, Chunyi Wen

**Affiliations:** ^1^Department of Biomedical Engineering, Faculty of Engineering, The Hong Kong Polytechnic University, Hong Kong, China; ^2^Developmental Biology and Neurogenetics, Technische Universität Darmstadt, Darmstadt, Germany

**Keywords:** acetylcholinesterase, bone development, bone homeostasis, bone degeneration, osteoporosis, non-enzymatic

## Abstract

Acetylcholinesterase (AChE), an enzyme catalyzing the degradation of acetylcholine, plays an important suppressive role in the cholinergic regulation by terminating the action of acetylcholine. The expression of acetylcholinesterase and other cholinergic components is not restricted to only brain and nerve tissues but can also be found in non-neuronal tissues like the immune system and bone tissue. Primary identification of these components has been achieved. However, the information about their specific functions and underlying molecular mechanisms in bone remains scattered. Here, the physiological process of bone development, homeostasis, and degeneration are introduced. Next, the cholinergic system and its expression in bone tissue is documented. Among them, special attention goes to AChE, as the structure of this enzyme suggests diverse binding affinities, enabled by a peripheral site and a catalytic site. The peripheral site supports the non-enzymatic function of AChE in non-neuronal systems. Based on recent studies, the non-neuronal roles of acetylcholinesterase, both enzymatically and non-enzymatically, in bone development, homeostasis and degeneration are summarized briefly together with potential mechanisms to support these functions. We conclude that AChE may be a potential therapeutic target for bone diseases like osteoporosis.

## Introduction

The parasympathetic nervous system is an important branch of the autonomic nervous system. It will be activated during rest and can be seen as “the brake.” It is related to sexual arousal, salivation, lacrimation (tears), urination, digestion, and defecation. Its action is complementary to that of the sympathetic nervous system. The parasympathetic nervous system is involved in the maintenance of the metabolism and is a cholinergic system. The neural cholinergic system comprises acetylcholine and its synthesizing and degrading enzymes, receptors, and transporters. The components of the cholinergic system are not only expressed in the nervous system but can also be found in other organs like bone (En-Nosse et al., 2009).

Acetylcholinesterase (AChE), the classic cholinergic hydrolase, is mostly known for degrading acetylcholine in the neural synapses. However, AChE is also involved in regulating bone development and remodeling. Pharmaceutical suppression of AChE has been associated with decreased risk of hip fractures, enhanced healing of osteoporotic fracture and reduced overall mortality in Alzheimer's patients (Tamimi et al., 2012; Ogunwale et al., 2020).

In this review, first the process of bone development and remodeling will be described. Then, the expression of AChE and other cholinergic components in bone cells or tissues will be summarized based on a thorough literature review. Followed by the key findings on the plausible role of AChE in bone development, homeostasis and degeneration, both enzymatically and non-enzymatically, at the crossroads between the latest clinical epidemiology and molecular biology (**Table 2**). To conclude, current and emerging therapeutics for osteoporosis targeting AChE will be highlighted.

## Bone Development, Homeostasis, and Degeneration

Bone is well-known for its supporting, protective and locomotive functions in the human body. It is a multifunctional organ that plays a central role in many important biological processes. For instance, bone is a reservoir for calcium and phosphate which helps to maintain the metabolic homeostasis. It also houses and supports the hematopoiesis which is responsible for the production of immune cells. Bone has also been reported to have some new roles such as being an endocrine organ to regulate kidney (Fukumoto and Martin, 2009; Karsenty and Olson, 2016) and brain function (Rodan, 1998; Oury et al., 2013).

Two major processes are involved in bone development, namely intramembranous ossification and endochondral ossification. Intramembranous ossification refers to mesenchymal progenitors, which differentiate directly into osteoblasts. This occurs during the formation of cranial bones, parts of the mandible, and the clavicle. Briefly, the mesenchymal stem cells first form a template at the ossification center, then differentiate to osteoblasts and start secreting extracellular matrix and minerals, finally they harden and become bones.

The growth of the majority of bones, for example long bones, occurs in a relatively complex way (i.e., endochondral ossification). Generally, during endochondral ossification, first the cartilage forms at the growth sites, which is later replaced by mineralized bone tissue. This process starts with the mesenchymal stem cells differentiating to chondrocytes. The chondrocytes then start secreting hyaluronic acid, chondroitin sulfate, and collagen fibers to form a cartilage template of future bones. Under the regulation of a serial of bone developmental factors, the center of this hyaline cartilage starts to calcify. Without nutrients supply and with the accumulation of metabolic wastes, the chondrocytes gradually die to leave cavities in hyaline cartilage. These cavities allow blood vessels to penetrate and bring osteoblasts in, releasing minerals to replace the chondrocytes. The blood vessels also let osteoclasts in to create bone marrow. Osteoblasts and osteoclasts communicate persistently to moderate the bone structure. The thin plate of hyaline cartilage will exist throughout childhood and adolescence, between the diaphysis and epiphysis, known as the growth or epiphyseal plate (Olsen et al., 2000).

Even after the skeleton is mature, bone is constantly resorbed and replaced with new bone in a process known as bone remodeling. Bone undergoes the remodeling process in response to mechanical stimuli in daily life. This requires the orchestration of the bone formation and the bone resorption process, which is tightly controlled by bone-forming cells-osteoblasts and bone resorbing cells-osteoclasts. Physiological bone remodeling is necessary to repair damaged bone and to maintain mineral homeostasis. However, once the activity of osteoblasts-mediated bone formation is stronger than that of osteoclasts-mediated bone resorption, or the bone resorptive activity overwhelms bone formation, an imbalance, or says uncoupling occurs. This imbalance between bone formation and bone resorption leads to the appearance of bone diseases like osteoporosis (Yang et al., 2019).

Bone remodeling occurs in “basic multicellular units” (BMUs), which couples several types of cells with multiple factors and cytokines (Hauge et al., 2001). BMUs are covered by a canopy of cells believed to be bone-lining cells or osteomacs (Pettit et al., 2008). The bone remodeling process that occurs in a BMU is highly collaborative and orderly. Most of the time, these canopy cells are in a dormant status and the mature osteoblasts, T cells and B cells derived from bone marrow continuously inhibit osteoclastogenesis by releasing osteoprotegerin (Theoleyre et al., 2004). However, after receiving bone remodeling initiation signals (e.g., structural damages caused by mechanical strain or hormone changes as a result of the systemic calcium homeostasis regulation), the BMU becomes active. Structural damage will attribute to osteocytes apoptosis, which lead to a decreased local level of transforming growth factor beta which will reduce the control of osteoclastogenesis. Moreover, reduced serum calcium level lead to the releasing of humoral factor parathyroid hormone, therefore stimulates the parathyroid hormone receptor on preosteoblasts. Consequently the osteoblasts start to secret monocyte chemoattractant protein-1 and assemble osteoclasts precursors from the capillary blood vessels or nearby macrophage progenitors to the bone surface (Li et al., 2007b). Additionally, a reduced osteoprotegerin expression and increased level of macrophage colony-stimulating factor and receptor activator of NF-kB ligand (RANKL) (Chen et al., 2018) in osteoblasts promote osteoclast proliferation and differentiation. Mature osteoclasts land in the damaged site and form the Howship's lacunae. In this sealed space, osteoclasts release hydrogen ions and proteolytic enzymes, like cathepsin K, to degrade the mineralized bone matrix. Later, the exposed demineralized collagen is removed by unknown phenotype reversal cells, which activate the bone formation signal.

Osteocytes, receiving humoral and mechanical signals, decrease sclerostin expression resulting in Wnt-induced bone formation. When sclerostin expression returns to the original level, bone formation stops and minerals deposits (Raggatt and Partridge, 2010). The whole BMU goes back to the resting phase. This process occurs throughout the bone and maintains bone homeostasis. Apart from these well-known bone-regulating factors, evidence indicates that cholinergic components also play a role in the regulation of bone homeostasis.

## The Cholinergic System

The cholinergic system is an indispensable part of the human and animal body and has an important function in regulating metabolic activities (Weinstock, 1997) such as learning, memory, sleeping, and stress regulation. It comprises acetylcholine in combination with its synthesizing and degrading enzymes, transporters and receptors.

### The Neuronal Cholinergic System

Acetylcholine, mostly known for its role as neurotransmitter in the nervous system, was first identified by Otto Loewi in 1921 (Loewi, 1921). Acetylcholine is synthesized by the enzyme choline acetyltransferase (ChAT) starting from acetyl coenzyme A and choline (Oda, 1999). Once synthesized, acetylcholine is transported from the cytoplasm to the synaptic vesicles of the neurons by the VAChT (vesicular acetylcholine transporter) (Erickson et al., 1994; Bittner and Martyn, 2019). During neurotransmission, an action potential reaches the presynaptic axon terminal which will lead to depolarization and fusion of the vesicles with the cell membrane. This will result in the release of acetylcholine into the synaptic cleft, followed by the activation of the postsynaptic acetylcholine receptors. Two types of acetylcholine receptors are identified: the nicotinic and muscarinic receptors. Nicotinic acetylcholine receptors are pentameric ligand-gated ion channels (Fon and Edwards, 2001). So far 10 subtypes have been identified. These subtypes can be assembled homomerically (e.g., the pentameric α7 receptor) or heteromerically (e.g., the pentameric α4β2 receptor) using following five subunits: α, β, γ, δ, ε. They are present on the post-junctional membrane and are instantly activated by acetylcholine for signal transmission. The signal mediates the fast depolarization and excitation of the target cell by allowing the entry of Ca^2+^ (Dani and Bertrand, 2007; Chuang, 2010). The muscarinic acetylcholine receptors can be activated by muscarine or acetylcholine. They are G-coupled protein receptor complexes that are generally responsible for the slow recovery of target cells after stimulation. Five subtypes (M1-5) of the muscarinic acetylcholine receptor are identified, each of them with a slightly different function (Kramer, 2016).

Acetylcholinesterase (AChE) is a well-known enzyme that catalyzes the hydrolysis of choline esters, such as acetylcholine. After hydrolysis of acetylcholine, choline and acetic acid are formed (Rosenberry, 1975; Bittner and Martyn, 2019). AChE functions rapidly and efficiently in the neuromuscular synapses: 50% of acetylcholine molecules are hydrolyzed by AChE before they reach the postsynaptic receptor sites, and the other acetylcholine molecules are broken down by AChE after their activation of the acetylcholine receptors (Bittner and Martyn, 2019). AChE has thus an important suppressive role in the cholinergic regulation by terminating the action of acetylcholine. Next to AChE, another cholinesterase can also degrade acetylcholine and other esters. This enzyme is called butyrylcholinesterase (BChE) as it can quickly hydrolyze butyrylcholine. The tissues expressing AChE also always express BChE (Silver, 1974; Chatonnet and Lockridge, 1989; Darvesh and Hopkins, 2003). Although BChE is the dominant cholinesterase in plasma and liver, the expression and/or the activity of AChE is higher than that of BChE in the skeleton, muscle, brain, heart and placenta (Jbilo et al., 1994). Moreover, human individuals with hereditary complete absence of BChE activity are healthy and fertile (Manoharan et al., 2007). Additionally, BChE knock-out mice are fertile and have a normal phenotype unless challenged with drugs (Li et al., 2008). In contrast, total inhibition of AChE leads to death due to respiratory failure. AChE nullizygous mice are only able to live up to 21 days after birth with delayed physical development (Xie et al., 2000). In this review, we will focus on discussing AChE as this enzyme is more necessary for a normal physical development.

### Non-neuronal Expression of Cholinergic Components in Bone System

The cholinergic components not only exists in neural tissues but are also widely distributed in many non-neural tissues. The non-neuronal expressed cholinergic components are denominated as the *non-neuronal cholinergic system* (NNCS). The presence of the non-neuronal cholinergic system is implied when acetylcholine is present or thus when cells have the capability of synthesizing acetylcholine and releasing it. The expression of acetylcholine receptors or acetylcholinesterase alone does not represent the NNCS. The NNCS is for example reported in human epithelial cells (Klapproth et al., 1997) and immune cells (Kawashima and Fujii, 2000) as acetylcholine, its synthesizing enzyme ChAT and its receptors are reported to be expressed in these cell types.

The NNCS in bone health and disease has gained an increasing interest as mounting evidence indicates its presence. This is summarized in Table 1. The mRNA of ChAT and VAChT is reported to be expressed in differentiated murine primary osteoblasts and MC3T3-E1 cells (Sato et al., 2010). The non-classical acetylcholine synthesizing enzyme carnitine acetyltransferase (CarAT) is identified in human SAOS-2 cells and mouse MC3T3-E1 cells (En-Nosse et al., 2009). Osteoclasts differentiated from murine bone marrow-derived macrophages also express ChAT (Mandl et al., 2016). At tissue level, ChAT has been identified in chicken embryo limbs (Spieker et al., 2016) and the mRNA expression of CarAT and VAChT has been confirmed in rat maxilla (Guo et al., 2014). The expression of ChAT or CarAT by osteoblastic and osteoclastic cells strongly suggests the local production of acetylcholine and thus the presence of the NNCS. Moreover, also the nicotinic and muscarinic acetylcholine receptors are expressed in osteoblasts, osteoclasts and bone tissues (En-Nosse et al., 2009; Sato et al., 2010; Liu et al., 2011; Kauschke et al., 2014; Ma et al., 2014; Ternes et al., 2015; Zablotni et al., 2015). The acetylcholine-hydrolyzing enzyme AChE is identified in mouse, rat and human primary osteoblasts (Inkson et al., 2004; Sato et al., 2010), and in both murine and human osteoblastic cell lines (Genever et al., 1999; Inkson et al., 2004). AChE is also detected in osteoclasts differentiated from murine bone marrow macrophages (Sato et al., 2015). Next to the expression in cells, a protein level of AChE is also expressed in rat maxilla, calvaria, femur, ulnae, and chicken embryo limbs.

**Table 1:** Expression of cholinergic components in bone cells or tissues.

**Bone cells or tissues**	**Study model**	**Expression of cholinergic components in bone cells or tissues**	**Expression level**	**References**
	**Osteoblastic lineage**			
Cells	Human SAOS-2 cell line; mouse MC3T3-E1 cell line	Carnitine acetyltransferase (CarAT)	mRNA	En-Nosse et al., 2009
	Differentiated Murine primary osteoblasts and MC3T3-E1 cells	AChE, vesicular acetylcholine transporter (VAChT), choline acetyltransferase (ChAT)	mRNA	Sato et al., 2010
	Mouse MC3T3-E1 cell line	AChE	mRNA	En-Nosse et al., 2009
	Human and rat primary osteoblasts, mouse MC3T3-E1 cell line, human MG63 cell line; human TE85 cell line	AChE	Protein	Inkson et al., 2004
	SAOS-2, MC3T3-E1, MG63, and TE85 cell lines; Primary rat osteoblasts	AChE	mRNA	Genever et al., 1999
	Primary cultured rat osteoblasts	AChE	Protein	Xu et al., 2017
	Human osteosarcoma HOS cells	Muscarinic acetylcholine receptors subtypes M1-5	mRNA	Liu et al., 2011
	Human Reaming debris derived mesenchymal stem cells (RDMSC) and osteoblasts differentiated from RDMSC	Muscarinic acetylcholine receptors subtypes M4, M5	mRNA	Zablotni et al., 2015
	Human SAOS-2 cell line	Muscarinic acetylcholine receptors subtypes M3, M5	mRNA	En-Nosse et al., 2009
	Mouse MC3T3-E1 cell line	Muscarinic acetylcholine receptors subtypes M1, M2, M4	mRNA	En-Nosse et al., 2009
	Differentiated Murine primary osteoblasts and MC3T3-E1 cells	Muscarinic acetylcholine receptors subtypes M1, M2, M4	mRNA	Sato et al., 2010
	Differentiated Murine primary osteoblasts and MC3T3-E1 cell	Nicotinic acetylcholine receptors α1, α6, α7, β4, δ, ε	mRNA	Sato et al., 2010
	Human Reaming debris derived mesenchymal stem cells (RDMSC) and osteoblasts differentiated from RDMSC	Nicotinic acetylcholine receptors α5, α7, α9	mRNA	Zablotni et al., 2015
	Human primary osteoblasts, MG63 osteoblastic cell line, human bone cores	Nicotinic acetylcholine receptor subunit α4	mRNA	Walker et al., 2001
	Human SAOS-2 cell line	Nicotinic acetylcholine receptors α3, α5, α7, α9, α10, β2	mRNA	En-Nosse et al., 2009
	Mouse MC3T3-E1 cell line	Nicotinic acetylcholine receptors α2, α5, α9, α10, β2	mRNA	En-Nosse et al., 2009
	**Osteoclastic lineage**			
	Osteoclast differentiated from murine bone marrow-derived macrophages	ChAT	Protein	Mandl et al., 2016
	Murine bone marrow macrophages derived from tibia and osteoclasts differentiated from murine bone marrow macrophages	AChE	Protein	Sato et al., 2015
	Osteoclast differentiated from human peripheral blood mononuclear cells	Muscarinic acetylcholine receptors subtypes M3	mRNA	Ternes et al., 2015
	Osteoclast differentiated from human peripheral blood mononuclear cells	Nicotinic acetylcholine receptors α2, α7	mRNA	Ternes et al., 2015
Tissues	Chicken embryo limbs	ChAT	mRNA	Spieker et al., 2016
	Rat maxilla	CarAT	mRNA	Guo et al., 2014
	Rat calvarias and femurs	AChE	mRNA and Protein	Xu et al., 2017
	Rat maxilla	VAChT and AChE	mRNA and Protein	Guo et al., 2014
	Chicken embryo limbs	AChE	Protein	Spieker et al., 2016
	Rat ulnae	AChE	Protein	Inkson et al., 2004
	Rodents femur bone	Muscarinic acetylcholine receptors subtypes M1-5 expressed in rat femur; M1, M4, M5 expressed in mouse femur	mRNA	Liu et al., 2011
	Bovine bone	Muscarinic acetylcholine receptor subtypes M2, M3, and M4 were detected in bovine blade bone; subtypes M2 and M3 were detected in spongy bone; subtypes M2 and M4 were identified in periosteum	mRNA	Liu et al., 2011
	Human rib	Muscarinic acetylcholine receptors subtypes M1-5	mRNA	Liu et al., 2011
	Rat thoracic vertebra	Muscarinic acetylcholine receptors subtypes M3, M5	mRNA	Kauschke et al., 2014
	Mouse tibia tissue	Muscarinic acetylcholine receptors subtypes M1, M2, M4, M5	mRNA	Ma et al., 2014
	Rat maxilla	Muscarinic acetylcholine receptors subtypes M1, M2, M3, M4, M5	mRNA	Guo et al., 2014
	Rat maxilla	Nicotinic acetylcholine receptorsα1, α2, α3, α5, α7, α10, β1, β2, β4, γ	mRNA	Guo et al., 2014

## Classic Enzymatic Function of Acetylcholinesterase in Bone Tissue

Literature investigating the role of the classical function of AChE in bone was summarized in Table 2. This data point to a role of non-neural AChE in bone development and postnatal bone remodeling.

**Table 2:** Function of AChE during bone development and homeostasis.

**Stage**	**Discovery**	**Study model**	**Enzymatic/non-enzymatic**	**Source of acetylcholine**	**References**
Intramembranous ossification	mRNA expression of acetylcholine, VAChT, AChE, nicotinic receptors and muscarinic receptors is identified in rat maxilla.	Rat	Enzymatic	Mesenchymal stem cells, bone matrix and bone marrow cells	Guo et al., 2014
	The AChE activity and protein amount first increases during embryonic period to the 6th days after birth and then decreases to a stale level in 2 months after birth in rat calvarias.	Rat	Enzymatic	N/A	Xu et al., 2017
Endochondral ossification	The AChE activity and protein amount first increases during embryonic period to the 6th days after birth and then decreases to a stale level in 2 months after birth in rat femurs.	Rat	Enzymatic	N/A	Xu et al., 2017
	The AChE activity first increases when hyaline cartilage forming and peaks at cartilage apoptosis stage, AChE activity decreases when the development direction shifts to bone mineralization. Implantation of acetylcholine- and ChAT-soaked beads benefits bone mineralization.	Chicken embryo	Enzymatic	Expression of ChAT indicates possible local production of acetylcholine	Spieker et al., 2016
	Genetic knockout of AChE, or BChE, or both of them accelerates cartilage remodeling into mineralizing bone.	Mouse	Enzymatic	N/A	Spieker et al., 2017
Postnatal bone homeostasis	16-weeks-old *Ache^+/−^* mice exhibited a reduction of the relative number of osteoclasts.	Mouse	Enzymatic	N/A	Kauschke et al., 2015
	Pharmaceutical suppression of AChE suggests a higher BV/TV ratio compare to control treatment of phosphate-buffered saline. AChE expression upregulates during osteoclastogenesis.	Mouse	Enzymatic	N/A	Sato et al., 2015
	Matrix coating with AChE promote osteoblastic cells attachment.	Rat; MC3T3-E1, MG63, and TE85 cell lines	Non-enzymatic	N/A	Genever et al., 1999
	Pharmaceutical suppression of AChE decreases the adhesion ability of osteoblastic cells without interfering their cell viability.	Human and rat primary osteoblasts, mouse MC3T3-E1 cell line, human MG63 cell line; human TE85 cell line	Non-enzymatic	N/A	Inkson et al., 2004

### Non-neuronal Cholinergic Role of AChE in Bone Development

Several studies focused on the non-neuronal cholinergic regulation of bone development.

Although the role of AChE during intramembranous ossification has not been fully understood, its expression has been identified in several rodent animal models, such as rat maxilla, which is mainly formed *via* intramembranous ossification (Guo et al., 2014). The expression pattern of AChE during embryonic and postnatal bone development has also been determined in rat calvarias (Xu et al., 2017), in which AChE activity and protein amount first increases during embryonic period to the 6th days after birth and then decreases to a stable level 2 months after birth. These studies suggest a potential regulative role of AChE in intramembranous ossification.

**Figure 1:**
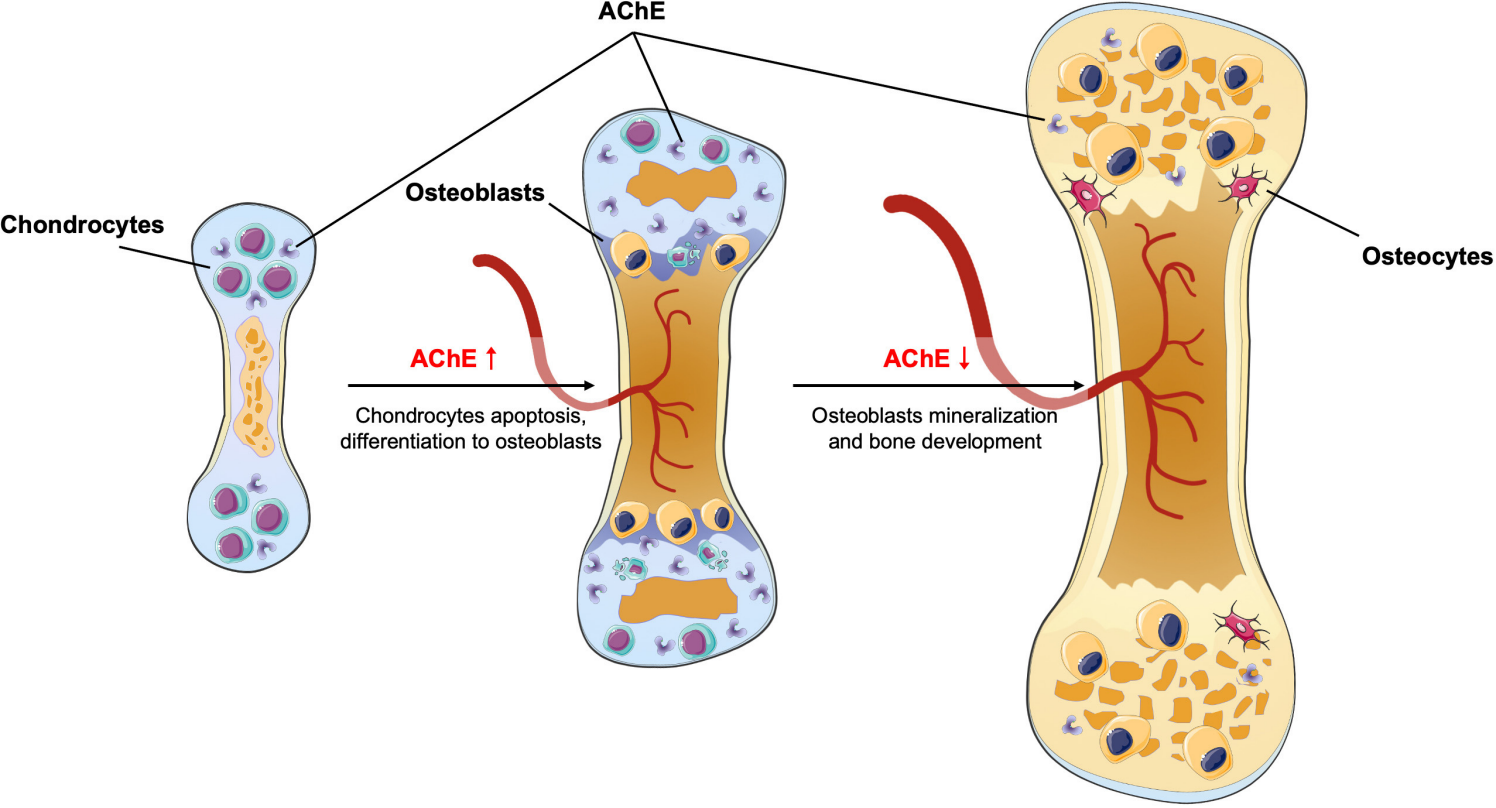
During endochondral ossification, AChE level first increases to favor chondrocytes apoptosis, later AChE level decreases and therefore accelerates bone mineralization.

The expression of AChE and ChAT during endochondral ossification is documented during early stages of embryonic bone development in both chicken, mice and rat animal models (Spieker et al., 2016, 2017; Xu et al., 2017). At the onset of early limb development, AChE is strongly expressed at hyaline cartilage, and is eventually elevated in apoptotic areas. In the chicken embryonic study, when the development direction shifts to bone mineralization, the expression of AChE decreased. Meanwhile, the AChE expression is slightly earlier than the that of ChAT in limbs, showing the leading role of AChE in endochondral ossification. The existence of ChAT suggests possible local acetylcholine production during embryonic bone development. These studies also showed that implantation of acetylcholine- and ChAT-soaked beads benefits bone mineralization (Spieker et al., 2016). In another study, the researchers analyzed endochondral ossification in mutant murine fetuses (Spieker et al., 2017), in which genes of AChE, or BChE, or both were deleted (called here A^−^B^+^, A^+^B^−^, A^−^B^−^, respectively). In all these mutant embryos, bone growth and cartilage remodeling into mineralizing bone were accelerated, although the source of acetylcholine is not directly investigated in this study, the researchers suggested that the acetylcholine might be expressed by mesenchymal stem cell in early endochondral ossification stage. The effect of AChE inhibition *in vitro* has also been confirmed, mesenchymal cells extracted from embryonic chick wing buds were used to perform micromass culture. Treatment of AChE inhibitor BW284c51 leads to decrease in cartilage and accelerated mineralization. In a rat animal model, the AChE activity in rat femur increased during embryonic day 18 to postnatal day 6, and later gradually decreased until 2 months after birth (Xu et al., 2017). All this evidence support that AChE—*via* balancing acetylcholine concentrations at a low level—can decelerate bone growth, thereby favoring cartilage apoptosis and hindering bone mineralization (Figure 1).

### Non-neuronal Cholinergic Role of AChE in Postnatal Bone Homeostasis

AChE is able to modulate postnatal bone homeostasis *via* its classic enzymatic role. 16-weeks-old *Ache*^+/−^ mice, who only express half of the brain AChE activity compared to their wild-type littermates, exhibited a reduction in the relative number of osteoclasts (Kauschke et al., 2015). Although the attenuation of AChE *via* genetic knock-out affects the systemic cholinergic system, this still suggests the possible non-neuronal function of AChE, that is, suppression of AChE might be beneficial for bone formation. This result was confirmed in a mouse animal model whereby pharmaceutical suppression of AChE suggests a higher BV/TV ratio compare to control treatment of phosphate-buffered saline. Moreover, after induction of bone loss in mice using RANKL, the AChE inhibitor donepezil could rescue the induced bone loss. The effects of AChE on bone formation and resorption were also verified *in vitro*. During osteoclastogenesis of bone marrow macrophages an upregulation of the AChE expression has been observed. Genetic knockdown of AChE *via* siRNA suppressed RANKL-induced osteoclast differentiation. In contrast, treatment of recombinant AChE protein upregulated expression of RANK, the receptor of RANKL, in bone marrow macrophages and thus osteoclast formation. Finally, also donepezil, an AChE inhibitor, could directly attenuate the osteoclastogenesis *in vitro*. Thus, the reduction of AChE leads to an increase of bone formation and reduction in bone resorption. The results of the study were also attributed to the increased acetylcholine activity (Sato et al., 2015).

## An Emerging Non-Neuronal and Non-Enzymatic Role of AChE in Bone Homeostasis

### Potential Multi-Tasking Protein—From the Molecule Structure of AChE to Its Function

AChE is a protein mainly known for its function to enzymatically degrade acetylcholine in the nervous system. However, as explained above, this enzymatic function is not limited to the nervous system but is also observed in non-neural systems for example in bone. Remarkably, its diversity of molecular forms and acetylcholine binding sites also suggests non-enzymatic functions in addition to its classical hydrolytic functions.

To exert this variety of functions in neural and non-neural systems, AChE presents a wide molecular diversity originating from a variation on the genetic, post-transcriptional, and post-translational level. Alternative splicing yields three variants of transcripts with the same catalytic domain but different C termini *via* the combinations of different exons: The “read-through” (AChE_R_), “hydrophobic (AChE_H_),” and “tail (AChE_T_)” transcript. The AChE_R_ transcript is translated into soluble monomers, which are mainly found in embryonic cells, cell cultures, and during stress conditions in the brain. AChE_H_ codes for glycophosphatidylinositol-anchored dimers, which are detected in hematopoietic cells. AChE_T_ is found in a variety of oligomeric forms (e.g., as homomers or heteromers). AChE_T_ is responsible for the regulation of the neurotransmission at the neuromuscular synapse. In this environment, AChE_T_ exists as a tetramer associated with either collagen-like Q subunit, or proline-rich membrane-anchoring protein (Massoulié et al., 1993; Sáez-Valero and Vidal, 1995; Massoulié, 2002; Xie et al., 2007, 2008, 2009, 2010; Leung et al., 2009; Mok et al., 2009; Dvir et al., 2010). It was discovered that AChE exists in osteoblasts as amphiphilic tetrameric globular form (AChE_T_) associated with the proline-rich membrane-anchoring protein I (Xu et al., 2017).

AChE is a multi-tasking protein with diverse acetylcholine binding sites: the catalytic site and a peripheral anionic site. The catalytic site is situated in a deep gorge of the enzyme and has two subsites: the ecstatic and anionic site. The ecstatic subsite is the catalytic site, resembling other serine hydrolases. This site consists of the catalytic triad: Ser203-Glu334-His447. The anionic subsite is the choline-binding pocket and interacts with the charged quaternary group of choline (Dvir et al., 2010). Competitive inhibitors bind on this catalytic site to prevent the action of AChE. However, other inhibitors will interact with an allosteric site, now known as the peripheral anionic site. This site is located near the entrance of the active site gorge. Binding of inhibitors will block the access to the catalytic site and/or modify the catalytic triad conformation (allosteric modification). In recent years, the peripheral anionic site was investigated for its role in non-enzymatic functions of AChE. This site could make the heterologous protein associations that affect cell recognition and adhesion functions (Bourne et al., 2003). A non-enzymatic role of AChE was already proven in neurogenesis and hematopoiesis. Studies showed that the addition of purified AChE could promote neurite growth from chick nerve cells in culture, while inhibitors targeting the catalytic site had no influence on this effect, suggesting a non-enzymatic action of AChE (Layer et al., 1993; Small et al., 1995). Next, blood cell progenitors could express AChE, regulating lymphocyte activation in both enzymatic and non-enzymatic manners (Paoletti et al., 1992; Lev-Lehman et al., 1997; Kawashima and Fujii, 2000).

### Non-enzymatic Function of AChE in Postnatal Bone Homeostasis

A non-enzymatic adhesion function is also assumed for AChE in postnatal bone growth. The idea emerged after the discovery of a new class of proteins: the cholinesterase-like adhesion proteins, for example, neurotactin, neuroligin, and thyroglobulin. These proteins have no catalytic activity, but their cholinesterase-like part can act as a protein-protein interaction domain. This domain is exposed extracellularly and can be used to form cellular junctions by binding other extracellular elements. The existence of these proteins leads to the assumption that acetylcholinesterase itself can act in protein-protein interactions (Botti et al., 1998; Scholl and Scheiffele, 2003).

To substantiate this interaction, it is crucial to define interaction partners for acetylcholinesterase. The first partner identified was laminin-1, more specifically the globular domain IV of the beta-1 chain. Next, also collagen IV was identified as a possible partner. AChE binds to these extracellular matrix components *via* its peripheral anionic site as inhibitors of the peripheral anionic site (fasciculin, propidium, and gallamine) interrupt these binding. This interaction is mainly electrostatic, as it is influenced by ionic strength and pH (Johnson and Moore, 2003, 2004; Paraoanu and Layer, 2008).

The adhesion of AChE with extracellular matrix components allows cell-to-cell recognition or cell signaling *via* membrane receptors, suggesting an important role in bone homeostasis (Johnson and Moore, 2003, 2004; Paraoanu and Layer, 2008). Moreover, Wnt-3a and RunX-2 could modulate the transcription of AChE mRNA variant 3' terminated with exon 6 that encodes the catalytically and morphologically E6-AChE expression (Xu et al., 2016; Xu et al., 2017).

Most studies focusing on the non-enzymatic functions of AChE in bone investigate its role in the osteoblastic lineage. Osteoblasts express AChE as membrane protein and bone matrix protein (Genever et al., 1999). The latter requires excretion of the glycosylated form of AChE *via* the ER/Golgi apparatus pathway. AChE is detected at sites of bone formation, along cement lines, and in osteoid seams. It is hypothesized that differentiated osteoblasts secrete AChE at newly resorbed surfaces, which helps them attach. This leads to the formation of an AChE-rich cement line. The attachment of osteoblasts to AChE is confirmed by the addition of AChE inhibitors which leads to a reduction in cell adhesion. As bone formation proceeds, AChE gets trapped into the osteoid. Moreover, in further differentiated osteoblasts more non-secreted glycosylated AChE was formed, which contribute to bone contribute to bone matrix mineralization (Genever et al., 1999; Inkson et al., 2004; Vogel-Hopker et al., 2012).

Literature on the non-enzymatic functions of AChE in the osteoclastic lineage is limited. In one study, the researchers use heat-inactivated recombinant AChE to treat bone marrow macrophages. The treated cell culture demonstrated a higher number of TRAP positive cell without statistically significant differences (Sato et al., 2015). Future studies should focus more on the non-enzymatic role of AChE in the osteoclastic direction.

## Clinical Implications—AChE Inhibitors in Osteoporosis

Bone is an organ that is renewed constantly. For a healthy skeleton it is essential that bone formation and bone degradation are perfectly coordinated. In case of an imbalance of bone formation or degradation, diseases occur associated with bone loss (i.e., osteoporosis), or excessive formation of new bone as is occurring in osteopetrosis.

**Figure 2:**
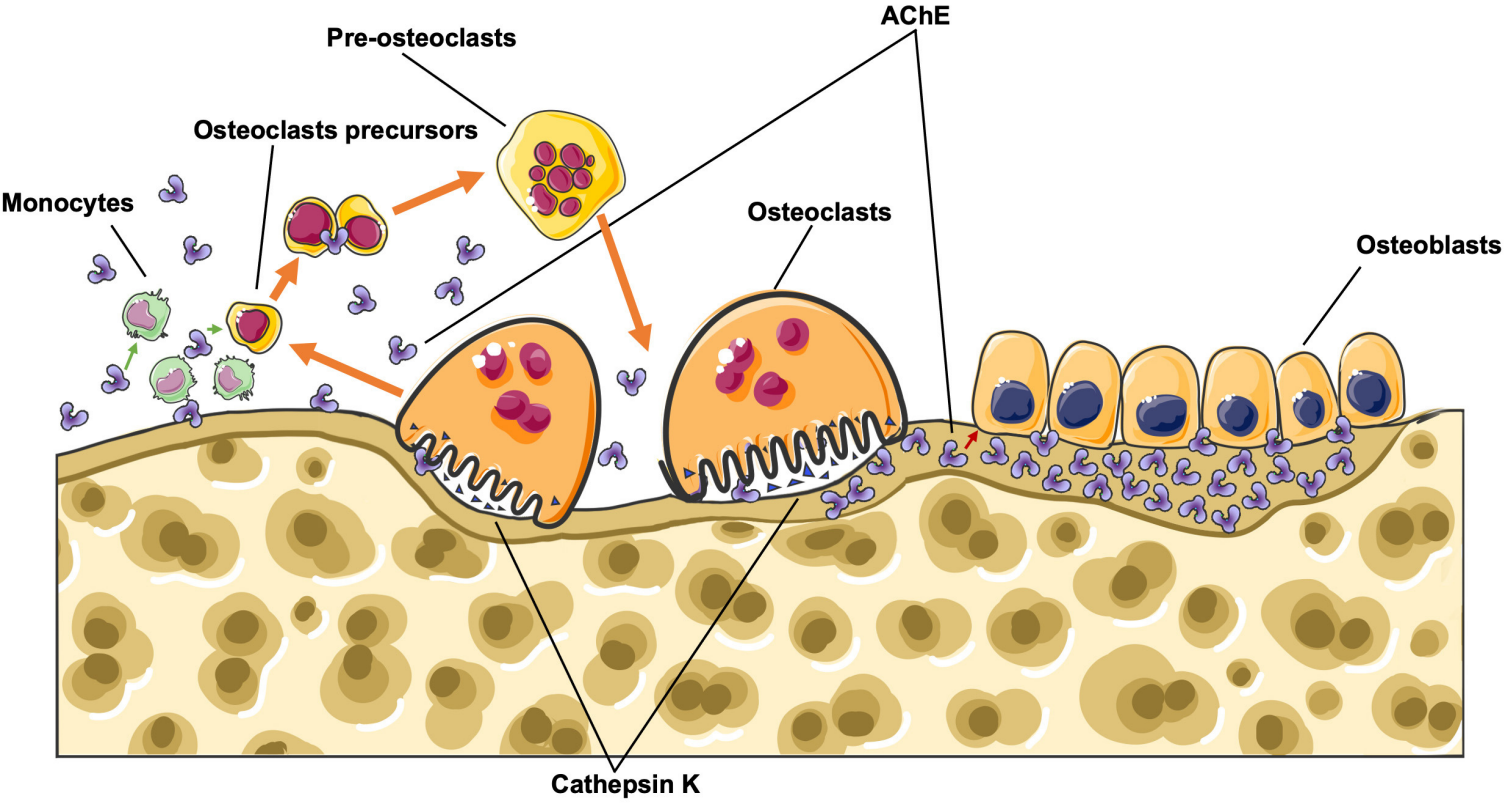
During bone remodeling, AChE is secreted by osteoblasts as adherent protein and is trapped in bone matrix during bone mineralization, when osteoclasts digest the bone matrix, AChE will be released to the surroundings, and therefore promote osteoclastogenesis.

Osteoporosis is a degenerative disorder marked by low bone density and microarchitectural deterioration of bone tissue (Marcus, 1996), which is caused by the uncoupling of bone formation and bone resorption, leading to huge medical and economic burdens to society (Bonjour et al., 2004; Foundation, 2009). Although primary understanding of this pathological process has been achieved, the underlying molecular mechanism remains unknown. Additionally, current prevention and treatment strategies (e.g., calcium and vitamin D supplement, bisphosphonates, parathyroid hormone) have limited effects, or unavoidable side effects (Kneissel et al., 2001; Jackson et al., 2006; Riek and Towler, 2011; Saita et al., 2015). Therefore, new approaches to control or treat this disease need to be found. As mentioned before, AChE regulates bone development and homeostasis in both acetylcholine-dependent and -independent manners (Figures 1, 2). Briefly, in the adult skeleton remodeling, AChE is secreted by osteoblasts as adherent proteins and is trapped in bone matrix during bone mineralization, when osteoclasts digest the bone matrix, AChE will be released to the surrounding environments, promoting osteoclastogenesis. Therefore, using an AChE inhibitor, which acts as well on the enzymatic pathway as the non-enzymatic pathway, might be interesting.

This indicates the necessity of dual blockade of AChE (the non-enzymatic as well as the enzymatic functional sites) in order to achieve stronger anti-catabolic effects on bone. For dual blockade of AChE, the dimerization of available drugs can be an effective strategy as this is a good approach to develop novel multifunctional drugs (Carlier et al., 1999, 2000; Li et al., 2007a). Huperzine A is a potent AChE inhibitor originally isolated from the Chinese medicinal herb *Huperzia Serrata* (Li et al., 2007a). Huperzine A has been approved as Alzheimer's therapy in China due to its specific anti-AChE activity. This component was reported to combine with itself to form bis(n)-hupyridone, or with previously FDA-approved anti-Alzheimer's drug tacrine to form hupyridone(n)-tacrine. Finally, the homodimer bis(n)-cognitin has been developed, synthesized as resulting from a computer model-based optimization strategy (Pang et al., 1996). Pharmacokinetic studies demonstrate that these dimers could be well-absorbed and readily cross the blood-brain barrier, suggesting that they might become applicable as drugs for both peripheral and central disorders (Yu et al., 2008), and could also represent emerging drug candidates for the treatment of osteoporosis.

## Conclusion and Future Directions

As documented before, during bone development AChE apparently accumulates during chondrocyte remodeling and apoptosis, while its expression decreased during the following bone mineralization. In bone homeostasis, bone remodeling is regulated by acetylcholinesterase based on both enzymatic and non-enzymatic mechanisms. Classically, AChE as a hydrolytic enzyme will decrease acetylcholine concentrations, suppressing bone formation and increasing bone resorption. Acting non-enzymatically, AChE as an adhesion protein will be secreted by, and adhere to osteoblasts at newly resorbed surfaces. The secretion will decrease as the bone mineralization process proceeds. Osteoclast formation is also stimulated by AChE *via* enzymatic and non-enzymatic pathways.

**Figure 3:**
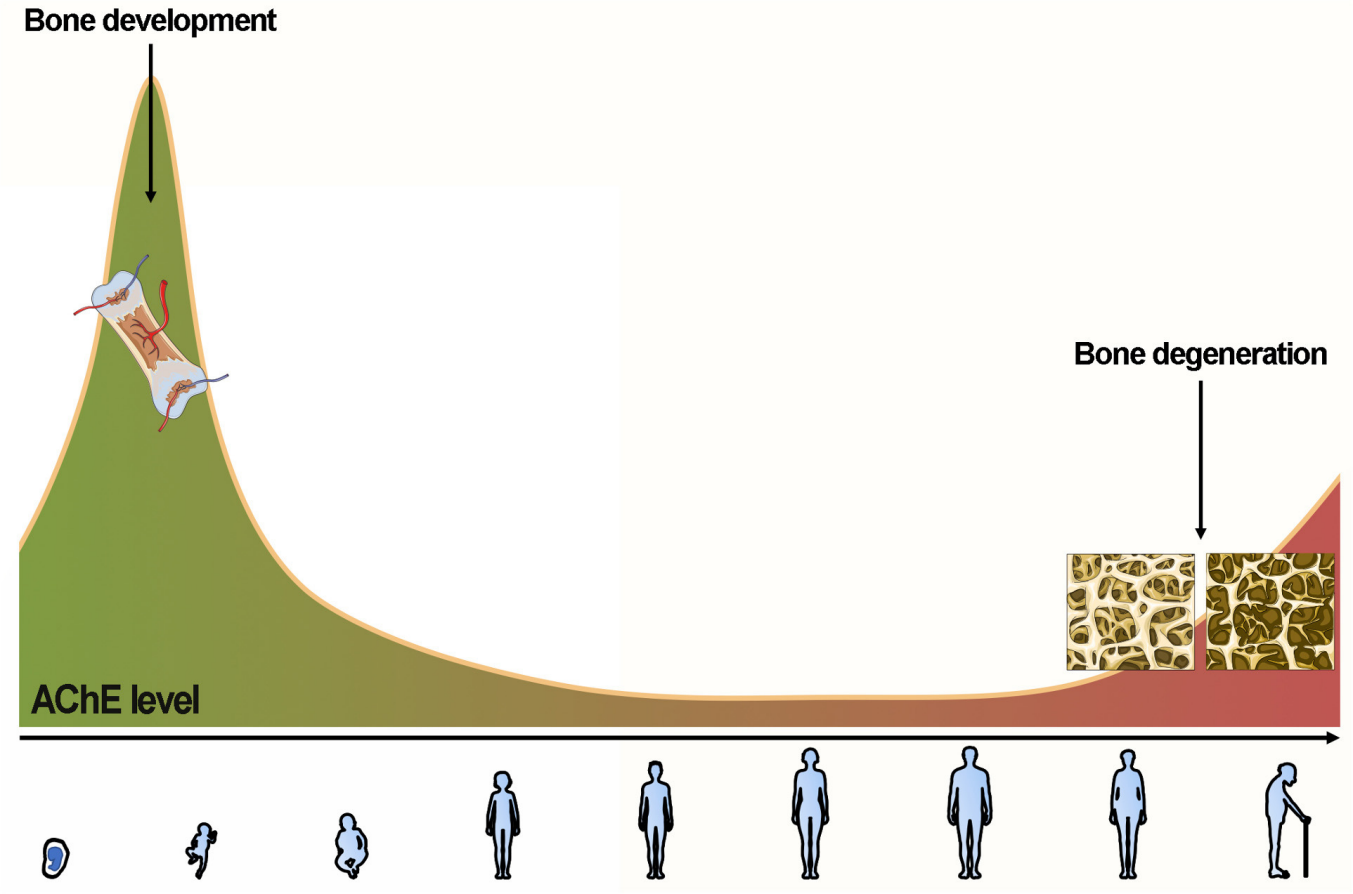
AChE first increases to favor chondrocyte remodeling to osteoblasts and gradually decreases and maintains a low level for a long time. AChE paradoxically increases after menopause or remains unchanged with aging. The (relative) increase of AChE contributes to age-related bone degeneration.

Normally, after adolescence, AChE remains at a lower but stable level compared to the level at the beginning of bone growth. The neuronal cholinergic activity decreases with aging (Perry, 1980), while the AChE expression level paradoxically increases after menopause (Newhouse and Dumas, 2015), or remains unchanged with aging (Namba et al., 1998). Thus, as a net result, the expression level of AChE is relatively increasing with aging, which contributes to age-related bone degeneration. Therefore, our summarized identification of previous studies suggests an important regulative role of AChE in skeletal systems (Figure 3).

Even though there is substantial evidence in literature indicating the importance of non-neuronal cholinergic regulations of bones, there is still much to learn about the complex relationship between the cholinergic system and bone, especially related to non-enzymatic action patterns. Future studies are required in order to investigate whether the peripheral anionic site acts as a protagonist for the non-enzymatic functions of AChE protein. Another area that needs investigation is the mechanism by which AChE affects the interactions of osteoblasts and osteoclasts. Although we know that AChE regulates proliferation and differentiation of both osteoblasts and osteoclasts, respectively, its effects on interactions of osteoblasts and osteoclasts leave relevant questions wide open.

## Author Contributions

CW and XL proposed the idea of this review and developed and interpreted the figures. XL and ML draft the manuscript. PL and CW extensively revise the manuscript. All the authors revised and approved the final version of the manuscript.

## Conflict of Interest

The authors declare that the research was conducted in the absence of any commercial or financial relationships that could be construed as a potential conflict of interest.

## Abbreviations

AChE: acetylcholinesterase
RANKL: receptor activator of NF-kB ligand
BMU: basic multicellular unit
ChAT: choline acetyltransferase
VAChT: vesicular acetylcholine transporter
BChE: butyrylcholinesterase
NNCS: non-neural cholinergic system
CarAT: carnitine acetyltransferase.
